# Meeting Reports

**DOI:** 10.1155/S146392468000048X

**Published:** 1980


acid method has been accepted as a satisfactory procedure for analysing fructose as a measure of enzyme activity [12], therefore the present improved automated procedure presented here should be similarly acceptable for measuring fructose from a wide variety of sample matrices.

REFERENCES

[1] Wardrip, E. K., *Food Technol. (London)*, 1971 **25**, 5, 47.
[2] Tsumura, N. and Sato, T., *Agri. Biol. Chem. (Japan)*, 1965 **29**, 2, 1123.
[3] Takasaki, Y., *Agri. Biol. Chem. (Japan)*, 1966 **30**, 2, 1247.
[4] Jermyn, M. A., *Anal. Biochem.*, 1975 **68**, 332.
[5] Halhoul, M. N. and Kleinberg, I., *Anal. Biochem.*, 1972 **50**, 337.
[6] Nixon, D. A., *Clin. Chim. Acta*, 1969 **26**, 167.
[7] Shahidullah, M. and Khorasani, S. S. M. A., *Anal. Chim. Acta*, 1972 **61**, 317.
[8] Zender, R. and Falbriard, A., *Clin. Chim. Acta.*, 1966 **13**, 246.
[9] Dische, Z. and Borenfreund, *J. Biol. Chem.*, 1951 **192**, 583.
[10] Dische, Z., *Methods in Carbohydrate Chemistry*, 1962 **1**, 481.
[11] Cadmus, M. C. and Strandburg, G. W., *Anal. Biochem.*, 1968 **26**, 3, 484.
[12] Lloyd, N. E., Khaleeluddin, K. and Lamm, W. R., *Cereal Chemistry*, 1972 **49**, 544.
[13] Nakamura, M., *Agri. Biol. Chem.*, 1968 **32**, 6, 701.
[14] Kainuma, K., Tadokoro, K., Sugawara, K. and Suzukis, S., *Dempun Kogyo Gakkaishi*, 1967, **15**, 1, 10, *Chem. Abstr.* 1968 **69**, 83953.
[15] Simons, S. S. and Johnson, D. F., *J. Org. Chem.* 1978, **43**, 14, 2886.

### Meeting Reports

### ECCLS discussion group meeting on instrument testing

At a recent meeting of the newly formed European Committee for Clinical Laboratory Standards (ECCLS) several *ad hoc* groups met to propose policies which this Committee might pursue. Groups met to cover the subjects of labelling, specimen collection, materials for quality control and quality control procedures. Reports from all groups are available to ECCLS Members from Irene Batty, ECCLS Executive Director, Wellcome Research Laboratories, Langley Court, Beckenham, Kent, England. The report from the Instrument Testing Group is reproduced below. It is emphasised that the proposals do not necessarily represent the policy of the ECCLS Board.

The Group was instructed to propose a policy which the Committee might pursue in the important area of instrument testing. Those present represented the three major participating sectors in the membership of ECCLS with three members from government, three from industry and seven from the professions. *

During a wide-ranging discussion the following conclusions were reached:

1. Work in the area of instrument evaluation should be given a high priority in the initial programme for the ECCLS. Evaluation protocols should be produced as quickly as possible and a scheme should be inaugurated for organising instrument evaluations to cover the member countries of ECCLS.
2. For the above purposes a Working Group should be formed with a membership coverage similar to that of the present *ad hoc* group but including a professional statistician.
3. The present replication of effort in this area is extremely wasteful in time and money both for industry and the profession and the resulting evaluations are all too often of a superficial nature.
4. Evaluations and any recommendations should be done and made by potential users. Specialists should look into certain specific areas, ie a photometer or a syringe system if present. Testing should be done to a parametric standard, ie the protocol should describe how the test is to be carried out and should not lay down limits.
5. Any previous testing done by the US National Committee for Clinical Laboratory Standards (NCCLS) or another organisation should be taken into account in arriving at conclusions. If instruments have been in use in laboratories prior to the testing scheme commencing, user experience should be canvassed.
6. A general protocol should be produced to cover all types of instruments for all specialties within pathology. This would then be divided into sections including for example (a) electrical safety (this must comply with the rules in individual countries), (b) haematology, (c) clinical chemistry. Clinical chemistry and haematology would then be broken down for various types of instruments, ie for clinical chemistry – flame photometers and colorimeters. Several instruments (eg colorimeters) are common.
7. Each instrument will inevitably require a slightly different approach and the ECCLS must therefore determine a revised protocol applicable to a particular instrument before testing is commenced.
8. Special arrangements should be made for instruments specifically constructed for use in doctors' surgeries, wards, etc.
9. Before every evaluation a contract should be carefully drawn up between the producer and the testing organisation. The evaluation process should be done as quickly as possible. All instruments tested must be production models. No prototypes should be tested in the proposed scheme. Testing should always be done against a carefully prepared protocol and there should therefore be no chance of litigation by the producer.
10. The overall arrangements must include a means whereby the producer can comment upon the final report, and his comments should be published with the report.
11. The ECCLS Group would ideally pick three routine laboratories in at least two different countries where testing should take place simultaneously. In each laboratory at least three different technicians should be involved with operating the instrument on their own if possible. It may not be practical to install three large instruments at one time and arrangements may be modified to cover this difficulty.

### Implementation

Professor Haeckel (Hannover) together with a selected group in Germany has produced a protocol at the instigation of the IFCC Expert Panel on Instrumentation. The German Group is currently using the protocol in an instrument programme and in the light of experience so gained, it will be modified and subsequently tested again jointly with a French Group. This protocol could form the basis of an ECCLS document. It is hoped that a final version should be available in the summer of 1980 and thereafter no time should be lost in the ECCLS taking it up for use. The IFCC Expert Panel would endeavour to obtain full international acceptance

**Membership of Instrument Testing Group:-*
*P Bonini, Karl-Georg von Boroviczeny, Christian Collombel, J Dautlick, E J Dixon, F Esser, T Gerritsen, J Bierens de Haan, W Jansen, G Merry, F L Mitchell (Chairman), M Roth, D B Slade.*

of the ECCLS protocol either as such or in modified form.

To cover the costs of each evaluation, it is proposed that the manufacturer should loan, install and maintain instruments in the testing laboratories throughout the trial. The actual costs of the trial should not be born by the instrument manufacturer, thereby ensuring independence for the testing procedure. An endeavour should be made to pool finances currently used by governments etc for testing purposes.

The final report, whether favourable or not, should be made widely available and consideration should be given to the sale of reports. It would be hoped that users in all member countries of ECCLS would accept the results of instrument testing using the suggested arrangements.

Dr Esser of the Physikalisch Technische Bundesanstalt, West Berlin, is currently gathering information on evaulation procedures in other countries and hopes to be able to make this information available as a data base for ECCLS. He also offered to use his good offices in an endeavour to rationalise finance for activity in the area.

*F. L. Mitchell*

### High speed automatic analysis

A joint meeting of the Analytical division of the Chemical Society and the Association of Clinical Biochemists was held at Strathclyde University, Glasgow on May 30th 1980 under the title "High Speed Automatic Analysis".

The papers presented reflected the two basic approaches to automation; ie continuous flow methods versus automated batch analysis methods, and the organisers arranged the programme so that the morning session consisted of papers describing Flow Injection Analysis (FIA) techniques, whilst during the afternoon session various automated batch methods were presented. As might be expected at a joint meeting of this type there was a strong bias towards analyses of clinical importance in most of the papers given.

After introductory remarks from Dr Gordon Cochrane, chairman of the Scottish Region, the first session of the meeting took place under the chairmanship of Mr Derrick Porter of the Laboratory of the Government Chemist. The first paper was given by Dr J. Ruzicka of the Technical University of Denmark, who outlined the principles of FIA and went on to describe the way in which this technique could be adapted to suit a wide range of analyses. He included numerous examples in his talk, and showed results which demonstrated the extremely high sample throughput possible by FIA.

This was followed by a paper from Dr E.H. Hansen, a colleague of Dr Ruzicka who described some recent developments in FIA based on the use of potentiometric and spectrophotometric flow-through detectors.

Among the many topics covered by Dr Hansen in his talk were the development of microelectrodes to enable measurements of pH, pCa etc to be made accurately in a flowing stream using samples of only a few microlitres, and the use of merging-zone FIA to reduce reagent consumption to only a few microlitres per sample for analyses requiring very expensive reagents, such as certain enzymes.

After a short break for coffee, the session resumed with a paper given by T.J. Sly describing a system under development at University College Swansea which utilises FIA as a post-column detector for reducing sugars separated by HPLC. The system employs a sampling valve to inject samples of column effluent at regular intervals into a stream of a colour-forming reagent, and the sugar concentration is detected using a novel flow-through photometer which was described in some detail.

The final paper of the morning session was given by Dr J.N. Miller who presented a number of FIA systems developed at Loughborough University using fluorescence and luminescence detectors, and described how these techniques could be applied to the determination of albumin in serum using an energy-transfer immunoassay, and the study of drug-protein binding parameters by fluorescence. Dr Miller showed how, in both the examples mentioned above, stopped-flow and merging-zone techniques had been employed to minimise reagent consumption.

After a pleasant lunch, the conference resumed for the afternoon session, which opened with a paper by Dr C.P. Price of Southampton General Hospital who reviewed the operating principles and design features of the centrifugal fast analyser, with special reference to its application in clinical analysis. Dr Price explained how the high speed scanning ability of the instrument had led to the development of kinetic assay techniques in clinical analysis, and illustrated his talk with slides showing the important features of several commercial instruments.

The second paper of the afternoon was presented by Mr A.G. Bufton of Technicon Instruments Ltd, who described the operation of the new Technicon Quantachem automated discrete analyser, currently undergoing evaluation by the DHSS. This machine represents something of a departure for Technicon who, having dominated the field of continuous-flow analysis for the last twenty years, are now entering the field of discrete analysers. Mr Bufton laid great emphasis on the ease of operation of the new machine, which features an on-board computer for control and processing of results.

This was followed by a break for tea, after which Dr F.L. Mitchell of the Clinical Research Centre, Harrow, presented the last paper of the conference which dealt with the development of the DACOS (Discrete Analysis with Continuous Optical Scanning) analyser system, an interesting machine which embodies many of the features of the centrifugal analyser in what is basically a conventional automated discrete analyser.

The system features an optical assembly rotating at high speed in the centre of a sample carousel, thereby allowing rapid scanning of the reaction mixtures without the limitation of having to change an entire batch of samples at once, as in the centrifugal analyser. Dr Mitchell then went on to describe the new Kodak Ektachem analysis system, and offered his views on the way in which this kind of technology would affect the job of the clinical chemist in the future.

The organising committee are to be thanked for having put together such an interesting meeting, and it is hoped that other joint meetings of this type will be organised in the future. Summaries of the papers presented at the meeting are to appear shortly in Analytical Proceedings.

*Tim Sly*

BIBLIOGRAPHY

[1] Haeckel, R. Sonntag, O. and Petry, K., *Journal of Automatic Chemistry,* 1979, 1, 5, 273.

